# Responsive Janus Structural Color Hydrogel Micromotors for Label-Free Multiplex Assays

**DOI:** 10.34133/2021/9829068

**Published:** 2021-11-20

**Authors:** Huan Wang, Lijun Cai, Dagan Zhang, Luoran Shang, Yuanjin Zhao

**Affiliations:** ^1^Department of Clinical Laboratory, Institute of Translational Medicine, The Affiliated Drum Tower Hospital of Nanjing University Medical School, Nanjing 210002, China; ^2^The Eighth Affiliated Hospital, Sun Yat-Sen University, Shenzhen 518033, China; ^3^State Key Laboratory of Bioelectronics, School of Biological Science and Medical Engineering, Southeast University, Nanjing 210096, China; ^4^Shanghai Xuhui Central Hospital, Zhongshan-Xuhui Hospital, and The Shanghai Key Laboratory of Medical Epigenetics, The International Co-Laboratory of Medical Epigenetics and Metabolism (Ministry of Science and Technology), Institutes of Biomedical Sciences, Fudan University, Shanghai 200032, China; ^5^Chemistry and Biomedicine Innovation Center, Nanjing University, Nanjing 210023, China

## Abstract

Micromotors with self-propelling ability demonstrate great values in highly sensitive analysis. Developing novel micromotors to achieve label-free multiplex assay is particularly intriguing in terms of detection efficiency. Herein, structural color micromotors (SCMs) were developed and employed for this purpose. The SCMs were derived from phase separation of droplet templates and exhibited a Janus structure with two distinct sections, including one with structural colors and the other providing catalytic self-propelling functions. Besides, the SCMs were functionalized with ion-responsive aptamers, through which the interaction between the ions and aptamers resulted in the shift of the intrinsic color of the SCMs. It was demonstrated that the SCMs could realize multiplex label-free detection of ions based on their optical coding capacity and responsive behaviors. Moreover, the detection sensitivity was greatly improved benefiting from the autonomous motion of the SCMs which enhanced the ion-aptamer interactions. We anticipate that the SCMs can significantly promote the development of multiplex assay and biomedical fields.

## 1. Introduction

Micromotors are small artificial devices that can cause spontaneous motion by converting the fuels or the externally supplied energy into propulsion [1–5]. Owing to the considerable potential in carrying out tasks for many fields such as biomedicine and environmental science, they have attracted many interests of researchers. Particularly, the micromotors in the use of sensing have attracted increasing attention [6–9]. Compared to the traditional sensing strategies, the micromotor-based platform can contact the target analytes more frequently to exhibit higher sensitivity and reduced assay time [10–12]. Although with much progress, most of these strategies are carried out by labeled detection, which relies on a complicated and time-consuming process. Besides, the micromotors are hard to assay several analytes simultaneously, which is necessary for many situations. Therefore, functional micromotors with multiplex label-free detection capability are still anticipated.

**Figure 1:**
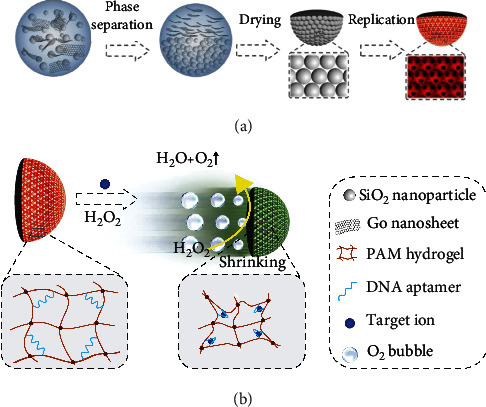
Schematic illustration of the fabrication and label-free ion detection of the SCMs. (a) Schematic illustration of the fabrication process of the SCMs. Droplets containing GO (loaded with magnetic and Pt NPs) and SiO_2_ nanoparticles are phase separated to form the Janus structural color particles, which were then replicated by hydrogels to obtain the SCMs. (b) Schematic diagram of the SCMs used for label-free ion detection in H_2_O_2_ solution. The NPs on the “dark section” of the SCMs catalyzed H_2_O_2_ to generate O_2_ bubbles and propelled the movement of the SCMs, and the aptamer on the “photonic section” captured the target ions and caused the color change due to shrinkage of the hydrogel scaffold.

In this paper, we present novel reduced graphene oxide (rGO) composited Janus inverse opal micromotors for multiplex label-free detection, as schemed in Figure 1. Inverse opals are materials with periodic macroporous structure, which are often negatively replicated from the artificial opals templates made of colloidal nanoparticles [13–17]. Owing to the ordered arrangement, inverse opals are endowed with apparent structural colors [18–23], which are beneficial for encoding [24–27]. Since the color depends on the microstructure, it is stable and free from the photobleaching and fluorescent background. Additionally, inverse opals can be modified with probes or functional sites, by which they can respond to the analytes and show the color change [28–31]. Therefore, the structural colors of the inverse opals can be used for sensing without further label strategies [32–34]. In contrast, graphene and its derivates are classic two-dimensional (2D) materials with an enormous specific surface area; so, they can be employed as carriers to load nanoparticles (NPs) with unique properties [35–40]. Particularly, when loaded with catalytic components, the graphene-based materials can generate bubbles to create propulsion [41–44]. Therefore, it is conceived that by integrating the sensing capabilities of inverse opals with the catalytic properties of graphene composites, a novel multiplex label-free assay platform could be well established.

Herein, we achieved this goal by the phase separation of the mixed solution containing graphene oxide (GO) and silica nanoparticles (Figure 1(a)). We employed a microfluidic device to generate droplets containing the hybrid solution. Because of the phase separation phenomenon in the droplets, Janus structural color particles with the two sections, respectively, enriched with GO and silica nanoparticles could be obtained after the water evaporation. By replicating the Janus particles with hydrogel functionalized with ion-responsive aptamers, the inverse opal particles were achieved. Platinum (Pt) and ferroferric oxide (Fe_3_O_4_) NPs were premixed with the GO solution and eventually loaded in the GO section of the Janus particles. The resultant inverse opal particles could convert the binding signal between aptamers and ions into the change of the structural color. This facilitated quantitative label-free analysis of multiple ions through structural color encoding. Besides, the presence of Pt NPs enabled the inverse opal particles to catalyze the decomposition reaction of H_2_O_2_ and generate bubble-induced propulsions, thereby acting as structural color micromotors (SCMs). Moreover, the Fe_3_O_4_ NPs provided a magnetic control of the micromotors' movement trajectory. This would largely improve the autonomous and directional movement ability and thus the detection efficiency of the SCMs. Overall, the present hydrogel Janus SCMs were excellent for label-free multiplex assays and will open new horizons in multiplex assays and micromotors.

## 2. Results

In a typical experiment, the Janus structural color particles were made by droplet templates containing silica nanoparticles and GO sheets generated from a microfluidic chip [45–52]. Pt and Fe_3_O_4_ NPs were immobilized on the GO sheets by simple adsorption, as shown in Figure S1 and Table S1. The SiO_2_ nanoparticles can disperse in water to form a transient homogeneous solution because of their electrostatic repulsions. However, there are many carboxyl groups on the GO sheets, which can be ionized in the mixed solution and reduce the pH of the solution. Hence, the adding of GO might cause the colloidal dispersion to become unstable. As a result, the SiO_2_ nanoparticles sedimented into the bottom of the droplets and the GO sheets enriched on the top due to a density difference. During the drying process, the droplet templates evolved into two layers gradually, and the GO sheets with Pt and Fe_3_O_4_ NPs were enriched in the upper part. It was noting that the mix solution need certain time to section into two layers, the slower the drying speed was, the more clear Janus structure the resultant particles would be with. After complete drying and calcination, the silica, in the bottom layer of the droplets, assembled into a close-packed crystalline structure. Because the GO sheets were reduced to rGO, the upper layer of the particles exhibited an inherent black color. In contrast, the bottom layer of the particles displayed brilliant structural color due to the presence of the silica colloidal photonic crystals. Therefore, the particles exhibited a Janus structure with a “dark section” hemisphere and another colorful “photonic section” hemisphere.

**Figure 2:**
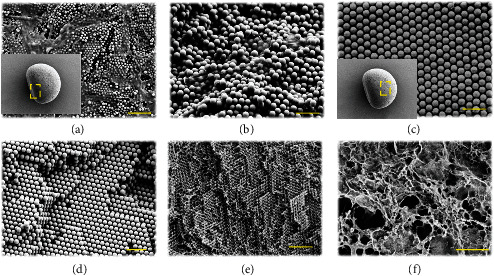
SEM images of the Janus structural color particle and the SCMs. (a, b). The surface (a) and inner (b) microstructure of the “dark section” (denoted by the dashed box in the inset image of (a)) of a Janus structural color particle. (c, d) The surface (c) and inner (d) microstructure of the “photonic section” (denoted by the dashed box in the inset image of (c)) of a Janus structural color particle. (e, f) The inner microstructure of the “photonic section” (e) and the “dark section” (f) of an SCM. Scale bars are 2 *μ*m in a and (e) and 1 *μ*m in (b)–(d) and (f).

The microstructure of the particles was characterized by scanning electron microscopy (SEM), as shown in Figure 2 and Figure S2. It was found that rGO largely presented on the surface and interior of the “dark section” (Figures 2(a) and 2(b)). It was found that there were many SiO_2_ nanoparticles in the “dark section.” However, in SiO_2,_ the nanoparticles in the “dark section” exhibited a short range order arrangement, which did not give rise to significant structural colors. On the contrary, rGO has strong light absorption capability, which rendered the “dark section” with a nearly black color. It was also worth mention that there were some GO sheets existed in the interstitial voids of the “photonic section.” However, the small size and amount of GO sheets had little influence on the assembly of the “photonic section.” Besides, the “photonic section” of the particles displayed a typical hexagonal close-packed (HCP) structure both at the surface and in the interior (Figures 2(c) and 2(d)). It was observed that there were many nanovoids between the silica nanoparticles. Therefore, when the Janus structural color particles were immersed in a pregel solution, the solution could fill the nanovoids of the particles rapidly due to capillary force. After polymerizing the pregel under irradiation of ultraviolet (UV) light, hydrogel was formed in the nanovoids, as shown in Figure S2a and S2b. The silica nanoparticles were then removed by sodium hydroxide (NaOH) etching to obtain the “inverse opal” hydrogel Janus structural color particles, whose microstructure was shown in Figure S2c. It was worth mentioning that due to the low mechanical strength, the hydrogel scaffold shrank and even collapsed during the drying process before observing by SEM. Hence, we employed the pregel with a higher ratio of crosslinker to construct the inverse opal particles. As expected, the porous structure was well preserved. Because the hydrogel particles were negatively replicated from the Janus structural color particles, they inherited the structural features of both the rGO “dark section” and the HCP “photonic section” (Figures 2(e) and 2(f)).

**Figure 3:**
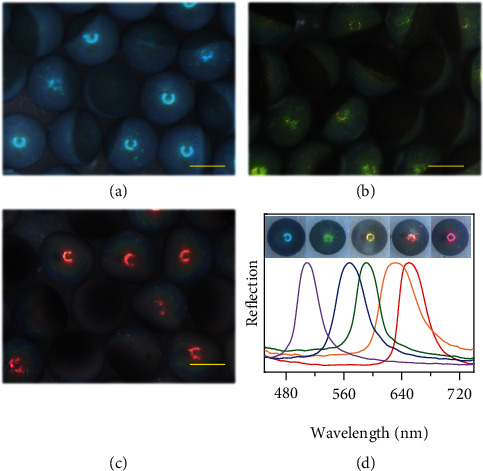
Optical characterization of the Janus structural color particles. (a)–(c) Reflection microscope images of three kinds of Janus structural color particles with blue (a), green (b), and red (c) colors. Scale bars are 200 *μ*m. (d) Reflective images and normalized reflective spectra of five different Janus structural color particles with distinct reflection peak positions.

The Janus structure often endows materials with more functions than homogeneous structure [53, 54]. In this study, owing to the ordered structure of the “photonic section,” the Janus structural color particles and corresponding inverse opal hydrogel particles possessed unique optical characteristics. Generally, such ordered structure results in the generation of photonic band gaps (PBGs), which prohibit the propagation of light with specific frequencies located in the gap. Therefore, both the Janus structural color particles and the hydrogel inverse opal particles exhibited brilliant structural colors and characteristic reflection peaks. The reflection peak positions *λ* of the particles under normal incidence can be estimated by integrating the Bragg Law and Snell Law:

(1) $$\begin{array}{c} \lambda =1.633{dn}_{\text{average}} \end{array}$$

where *d* is the diameter of the silica nanoparticles or the macropores, and *n*_average_ refers to the average refractive index of the “photonic section.” In this study, *n*_average_ was constant because the components of the particles were kept the same. Consequently, the colors of the particles depended on *d*. By using silica nanoparticles with different diameters, Janus structural color particles with various colors and corresponding reflective peaks were prepared, as illustrated in Figure 3. It was noted that the rGO “dark section” enabled broadband light absorption and brought a series of optical effects to the “photonic section.” Therefore, the SCPs possessed superior optical properties.

We found that the initial concentration of GO in the droplet templates significantly influenced the size and shape of the resultant Janus particles. As shown in Figure S3, when the size (500 *μ*m) and the silica content (20% w/v) of the droplets were kept constant, the size of the resultant Janus particles increased with the concentration of the GO solution until reaching a maximum value of 3 mg/mL. Besides, the shape of the Janus particles also changed with the GO concentration. Although being spherical at the beginning, the droplets evolved into distinct shapes after drying. Specifically, at a low GO concentration, the “dark section” of the final Janus particles showed thin and oblate shapes. With the increase of the GO concentration, the “dark section” gradually became thickened and rounded, the same as that of the “photonic section” (Figure S4). Consequently, we can construct Janus particles with different shapes for specific demand.

**Figure 4:**
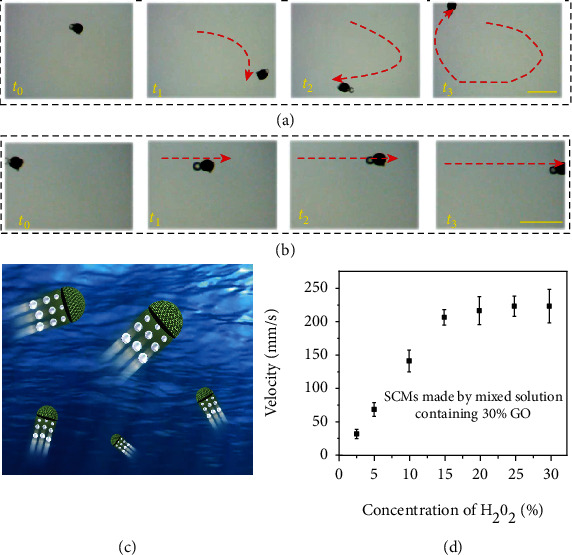
The movement capability of the SCMs. The movement of the SCMs without magnet guidance (a) and under magnet guidance (b). Scale bars are 1 mm. (c) Schematic diagram of the flexible movement of SCMs. (d) Plot of the movement velocity of the SCMs as a function of the concentration of H_2_O_2._ The number of replicates at any concentration was five.

Because the “dark section” contained enriched Pt NPs along with rGO, the Janus hydrogel inverse opal particle could generate a propelling drive when put in a solution containing H_2_O_2_. As confirmed in Figure 4(a) and Movie S1, it displayed autonomous movement propelled by the bubbles generated upon decomposition of H_2_O_2_ catalyzed by the Pt NPs. In this sense, it could serve as SCM. Besides, attributed to the enrichment of the Fe_3_O_4_ NPs, the movement of the SCMs can be regulated by an external magnetic field. As illustrated in Figure S5 and Movie S2, the SCMs moved directionally when a magnetic field was applied. Moreover, the two drive modes, i.e., the catalytic bubble drive and the magnetic drive can be integrated to achieve more precise control of the movement of the SCMs. As shown in Figure 4(b) and Movie S3, compared with the random movement propelled by only the bubbles, the SCMs displayed a highly controllable motion route under the integrated drive mode. These features endowed the SCMs with flexible motion abilities (Figure 4(c)). Based on this, we studied the movement speed of the SCMs as a function of the concentration of the fuel, that is, H_2_O_2_. We found that the velocity of the SCMs increased with the H_2_O_2_ concentration and reached a plateau at 15 vol% of H_2_O_2_ (Figure 4(d)).

Combining the merits of controllable motion and the intrinsic color tunability of the SCMs, we constructed an ion detection platform by functioning the SCMs with ion-responsive aptamers. Specifically, the hydrogel scaffold of the SCMs was composed of poly-acrylamide (PAAm). The amide groups on the hydrogel scaffold can be hydrolyzed to generate carboxyl groups, which could be further activated to conjugate with amino-modified DNA aptamers. The aptamers could be selected to respond to specific metal ions, and the binding between the ions and the aptamers could lead to the shrink of the hydrogel scaffold and the corresponding decrease of the macropore diameters. According to Equation (1), the reflective peaks of the SCMs should show a blueshift, which can be measured by a spectrometer. Hence, the SCMs could be used for label-free detection of metal ions simply by measuring the reflection spectra before and after interacting with ions.

**Figure 5:**
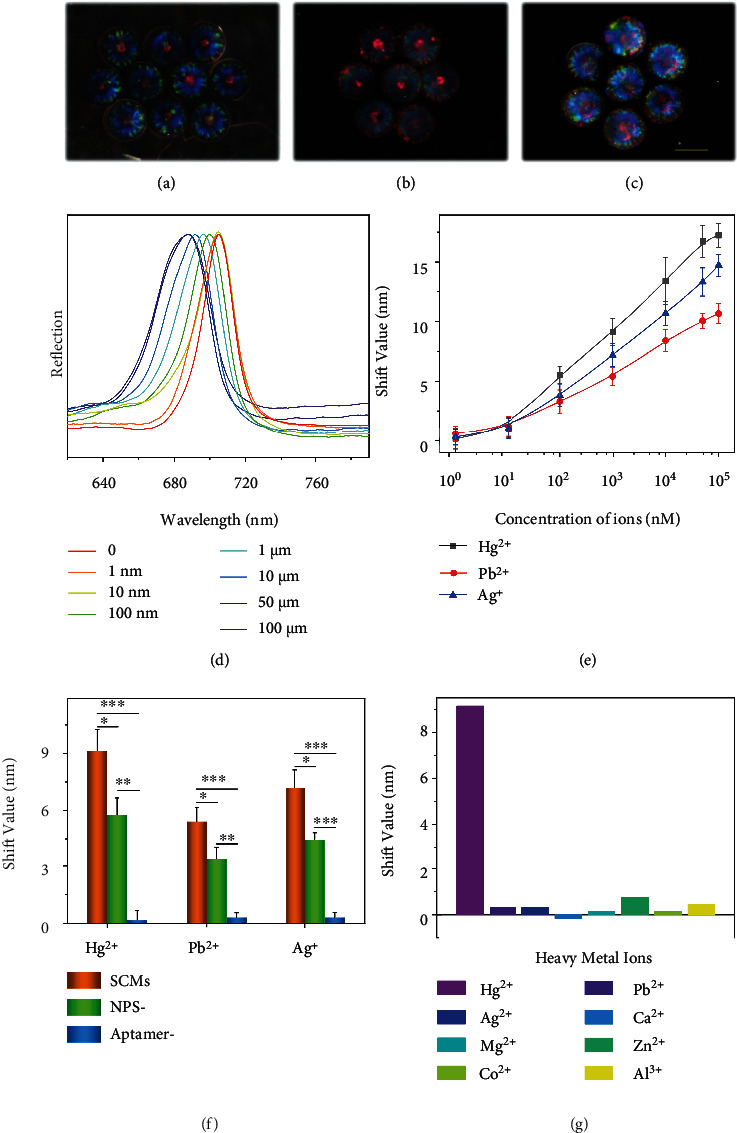
SCMs for ions detection. (a)–(c) Reflection images of the parent colloidal crystal structural color particles with crimson color (a), the corresponding inverse opal hydrogel SCM particles (b), and the SCMs functionalized with Hg^2+^-responsive aptamers (c). Scale bar is 200 *μ*m. (d) Optical response of the SCMs functionalized with Hg^2+^-responsive aptamers incubated in different concentrations of Hg^2+^ solution. (e) The relation between the reflective peak shift values of three kinds of aptamer-functionalized SCMs and the concentration of the target ions. (f) The reflection spectra shift of the aptamer-modified SCMs, Janus structural color hydrogel particles without Pt and Fe_3_O_4_ NPs (NP-), and SCMs without aptamer modification (aptamer-) in the corresponding metal ion solutions. ^∗^0.01 < *p* < 0.05, ^∗∗^*p* < 0.01, ^∗∗∗^*p* < 0.001. The number of replicates at any concentration was five. (g) The reflective peak shift values of the SCMs modified with Hg^2+^-responsive aptamers in various metal ion solutions (1 *μ*M for Hg^2+^ and 100 *μ*M for other ions), and the blank represents the SCMs treated with buffer.

To test this hypothesis, SCMs with crimson color were prepared and decorated with Hg^2+^-responsive aptamers. During this process, the SCMs maintained their intact shape, while the structural colors changed because of the alteration of the average refractive index (Figures 5(a)–5(c)). This was also true for orange-red-colored SCMs with Pb^2+^-responsive aptamers and green-colored SCMs with Ag^+^-responsive aptamers, as shown in Figure S6 and S7. It was worth mentioning that the volume ratio of the monomer and the crosslinker of the hydrogel scaffold were critical for the responsiveness of the SCMs. Generally, a higher value of the ratio results in pronounced shrinking because of more conjugate sites and a softer hydrogel scaffold. However, too high a value might cause the separation of the “dark section” and the “photonic section” due to their swelling discrepancy. Therefore, we optimized the value of this ratio at 2 : 1 in the following experiments. We then added the aptamer-functionalized crimson SCMs into the detection solution containing Hg^2+^ and H_2_O_2_. The SCMs moved in the solution by the propel of the generated bubbles. Consequently, the reflective spectra of the SCMs blue shifted after reacting with Hg^2+^, and the shift value increased with ion concentration, as shown in Figures 5(d) and 5(e). Similarly, the orange-red SCMs with Pb^2+^-responsive aptamers and green SCMs with Ag^+^-responsive aptamers could be used to detect Pb^2+^ and Ag^+^, as shown in Figure 5(e) and Figure S8. By contrast, the SCMs without aptamers did not show optical responses to the ions (Figure 5(f)). Since ions with high concentrations could not solve well in the solution with nearly neutral pH, hence we did not use high concentration ions, and the ion detection curves of the SCMs had not reached the saturation concentration. Besides, the shift value of the SCMs was higher than the hydrogel Janus structural color particles without incorporating the Pt or Fe_3_O_4_ NPs. This indicated that the autonomous movement of the SCMs increased the probability of the interactions of the aptamers and the ions, thereby achieving a higher sensitivity. Moreover, the SCMs exhibited significant shifts of reflective spectra only when exposed to the solution containing the target ions (Figure 5(g), Figure S9 and S10). In contrast, the optical changes of the SCMs in the solutions of noninteracting ions were negligible.

**Figure 6:**
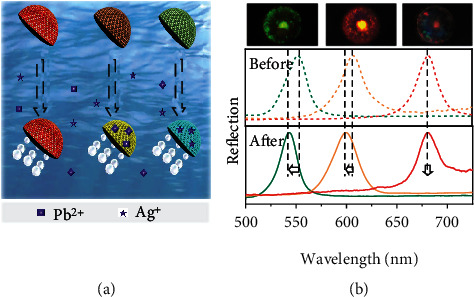
SCMs for multiplex ions detection. (a) Schematic illustration of the SCMs for multiplex label-free ion detection. (b) Reflective images (top) and reflective spectra (middle and bottom) of three kinds of SCMs before and after the multiplex assay. The crimson, orange-red, and green SCMs were functionalized with aptamer 1, aptamer 2, and aptamer 3, respectively. The dashed curves in the middle panel and the solid curves in the bottom panel are the reflective spectra of the SCMs before and after the detection, respectively.

We then conducted a multiplex assay for the detection of metal ions using SCMs, as illustrated in Figure 6(a). Briefly, three types of SCMs with crimson, orange-red, and green color were prepared, each of which were functionalized with distinct types of aptamer (denoted as aptamer 1, aptamer 2, and aptamer 3) that can bind with mercury ions (Hg^2+^), lead ions (Pb^2+^), and silver ions (Ag^+^), respectively (Table S2). The three kinds of aptamer-functionalized SCMs were mixed together and incubated in a solution containing Pb^2+^ and Ag^+^ (Movie S4). The reflective spectra of the SCMs before and after the incubation were shown in Figure 6. It could be found that only SCMs functionalized with aptamer 2 and aptamer 3 displayed blueshift of the reflective peaks, while those functionalized with aptamer 1 showed undetectable peak shift. Besides, each target ion could be identified tracing back to the initial reflective peaks of the SCMs, and their concentration could be estimated quantitatively by measuring the reflective peaks of the SCMs after detection. In other words, through a single-step spectral measurement, the decoding and detection could be realized at the same time. Overall, the SCMs are promising candidates for the simple, sensitive, and multiplex detections in different fields.

## 3. Discussion

In summary, we presented novel SCMs for label-free multiplex assays. Composite silica/GO microparticles were first fabricated from phase separation of microfluidic droplet templates, by which they formed a Janus structure with a “dark section” and a “photonic section.” The “photonic section” was composed of a silica colloidal crystal array that exhibited distinct structural colors, and the “dark section” consisted of GO doped with Pt and Fe_3_O_4_ NPs that could provide catalytic drive and magnetic drive. By using hydrogel functionalized with ion-responsive aptamers to replicate the colloidal crystal hemispheres, the SCMs could be prepared and used for label-free ion detection because the binding between ions and aptamers would lead to a blue shift of their characteristic reflective peaks. Besides, multiple ions could be detected simultaneously and differentiated based on the optical coding capacity of the SCMs. Moreover, due to their self-propelling property, the SCMs could realize autonomous motion and guided motion, which enhanced the detection sensitivity. These results manifested that the SCMs would play essential roles in multiplex analysis, micromotors, and related research areas.

## 4. Materials and Methods

### 4.1. Reagents

Octadecyltrichlorosilane (OTS), 2-hydroxy-2-methylpropiophenone (HMPP), acrylamide (AAm), N,N′-methylenebis (acrylamide) (Bis),N,N,N′,N′-tetramethylethylenediamine (TEMED), 2-(N-morpholino) ethanesulfonic acid (MES), and platinum nanoparticles (Pt NPs) were bought from Sigma-Aldrich (St. Louis, MO, USA). n-Hexane, nitric acid (HNO_3_), sodium hydroxide (NaOH), and ethanol were purchased from Sinopharm Chemical Reagent (Shanghai, China). N-Hydroxysuccinimide (NHS), 1-(3-dimethylaminopropyl)-3-ethylcarbodiimide hydrochloride (EDC), trimethylol aminomethane (Tris), and hydrogen peroxide (H_2_O_2_) were purchased from Aladdin Industrial Corporation, Shanghai, China. Magnetic nanoparticles (Fe_3_O_4_ NPs) were obtained from Nanjing Nanoeast Biotech Co., Ltd. Graphene oxide (GO) solution was bought from Nanjing XFNANO Materials Tech Co., Ltd. DNA aptamers were obtained from Sangon Biotech (Shanghai) Co., Ltd. Dimethyl silicone oil (with different viscosities) and glassware were purchased from Nanjing Wanqing Co. Ltd. SiO_2_ nanoparticles in different sizes were self-prepared. A Milli-Q Plus 185 water purification system (Millipore, Bedford, MA) was employed to provide ultrapure water with resistivity higher than 18 M*Ω*·cm.

### 4.2. Fabrication of Janus Structural Color Particles

Pt NPs, Fe_3_O_4_ NPs, and GO solution were mixed and stirred for 30 min. After centrifugation and washing, the NP-loaded GO was obtained. The SiO_2_ nanoparticle solution was then mixed with the NP-loaded GO solution. A group of the mixture was prepared with the concentration of the SiO_2_ nanoparticles set at 20 wt % regardless of the size, and the concentration of the GO solution varied from 0 to 5 mg/mL. Next, the mixture served as an inner phase fluid and flowed into a coflow microfluidic device, which resulted in the consecutive generation of droplets. The flow rates of the outer phase and the inner phase were 6 mL/h and 1 mL/h, respectively. The resultant droplets were collected and treated at 75°C overnight for the phase separation and the self-assembly of GO and SiO_2_ nanoparticles. Then, the dried particles were calcined for 4 hours in a tube furnace. The Janus structural color particles without NPs were prepared with the same procedure using the GO without NPs.

### 4.3. Characterization

The reflective spectra of the Janus structural color particles were measured by a fiber optic spectrometer (Ocean Optics, USB2000+) coupled with a metalloscope (Guangzhou Mshot Photoelectric Technology Co., Ltd.). The optical images were taken by a charge-coupled device (CCD) camera (Media Cybernetics EvolutionMP 5.0) coupled with a stereomicroscope (Nanjing Jiangnan Novel Optics Co. Ltd.). The microstructures were characterized by a scanning electron microscope (SEM) (Zeiss, Germany). Elemental analysis was carried out by an energy disperse spectroscopy (Oxford INCA).

### 4.4. Preparation of Structural Color Micromotors (SCMs)

A pregel solution (200 *μ*L) containing AAm, Bis, and HMPP (2 *μ*L) was employed to fill the nanopores of the Janus structural color particles (10 *μ*L) and then was polymerized by ultraviolet (UV) light. Then, the hydrogel composite particles were stripped from the bulk hydrogel and etched by NaOH solution to obtain the inverse opal Janus hydrogel particles, i.e., SCMs. The Janus hydrogel particles without NPs were prepared using the same procedure.

### 4.5. Preparation of DNA-Functionalized SCMs

Three kinds of DNA aptamers were dissolved into pure water to form the aptamer solution with a concentration of 500 *μ*M. The SCMs were treated with 0.1 M NaOH solution containing 10% TEMED for 1 h firstly. They were then washed and activated with EDC/NHS for 0.5 h. Afterwards, the SCMs reacted with the aptamer solution for 0.5 h at 37°C and were further incubated overnight at 4°C. After washing by buffer solution, the aptamer-functionalized SCMs were prepared successfully.

### 4.6. Movement of SCMs

The SCMs were immersed in the solutions with different concentrations of H_2_O_2_ (from 2.5 to 30%), and the movement was observed by a CCD camera. The trace of a single SCM particle was obtained by overlapping a series of time-lapse images.

### 4.7. Detection of Ions

The SCMs were added into the H_2_O_2_ solution (15%) with different concentrations of the target ions. Then, the SCMs were collected by an external magnetic field and washed by buffer solution. By measuring the reflective wavelengths of the SCMs and comparing them with the initial values, the relation between the concentration of the ions and the shift value of the reflective peaks of SCMs could be established. For the multiplex assays, three types of SCMs were functionalized with three kinds of aptamers and incubated in a solution containing only two kinds of target ions. After incubation, a buffer solution was employed to wash the SCMs at least three times. Finally, the multiplex detection result was determined by measuring the reflective wavelengths with a fiber optical spectrometer.

## Data Availability

All data are contained in the manuscript text and supplementary materials.

## Supplementary Materials

Supplementary 1 Figure S1: TEM characterization. Figure S2: SEM characterization of the particles. Figure S3: relationships between the diameter of the Janus structural color particles and the volume ratio of added GO. Figure S4: rReflection microscope images of Janus structural color particles with different volume ratio (5, 15, 25, 50%) of the GO solution (10 mg/mL) and colors. Figure S5: magnetic-induced directional movement of the SCMs under an external magnetic field. Figure S6: reflection image characterization of the SCMs made by colloidal crystal particles with orange color. Figure S7: reflection image characterization of the SCMs made by colloidal crystal particles with green color. Figure S8: optical response of the SCMs functionalized with Pb^2+^-responsive aptamers and Ag^+^-responsive aptamers in corresponding substrate solutions. Figure S9: the reflection spectra shift of the SCMs functionalized with Pb^2+^-responsive aptamers and Ag^+^-responsive aptamers in various metal ion solutions. Figure S10: the reflection spectra shift of the SCMs modified with Hg^+^-responsive aptamers, Pb^2+^-responsive aptamers, and Ag^+^-responsive aptamers in the target low concentration ion solution doped with high concentration other ions. Table S1: elemental analysis of the “dark section” of a Janus structural color particle. Table S2: sequences for the DNA aptamers.

Supplementary 2 Supplementary Movie. S1: SCM in H_2_O_2_ environment.

Supplementary 3 Supplementary Movie. S2: SCM movement under magnet guidance.

Supplementary 4 Supplementary Movie. S3: SCM in H_2_O_2_ environment with magnet guidance.

Supplementary 5 Supplementary Movie. S4: SCMs in H_2_O_2_ environment for multiple detections.
