# Supplementary material for: Responsive Janus Structural Color Hydrogel Micromotors for Label-Free Multiplex Assays
Source: Research (Wash D C). 2021 Nov 20;2021:9829068. doi: 10.34133/2021/9829068 (PMC8628110; doi:10.34133/2021/9829068)
Supplement: Supplementary 1 — Figure S1: TEM characterization. Figure S2: SEM characterization of the particles. Figure S3: relationships between the diameter of the Janus structural color particles and the volume ratio of added GO. Figure S4: rReflection microscope images of Janus structural color particles with different volume ratio (5, 15, 25, 50%) of the GO solution (10 mg/mL) and colors. Figure S5: magnetic-induced directional movement of the SCMs under an external magnetic field. Figure S6: reflection image characterization of the SCMs made by colloidal crystal particles with orange color. Figure S7: reflection image characterization of the SCMs made by colloidal crystal particles with green color. Figure S8: optical response of the SCMs functionalized with Pb2+-responsive aptamers and Ag+-responsive aptamers in corresponding substrate solutions. Figure S9: the reflection spectra shift of the SCMs functionalized with Pb2+-responsive aptamers and Ag+-responsive aptamers in various metal ion solutions. Figure S10: the reflection spectra shift of the SCMs modified with Hg+-responsive aptamers, Pb2+-responsive aptamers, and Ag+-responsive aptamers in the target low concentration ion solution doped with high concentration other ions. Table S1: elemental analysis of the “dark section” of a Janus structural color particle. Table S2: sequences for the DNA aptamers. [file 9829068.f1.docx]

Supporting Information

*Responsive Janus structural color hydrogel micromotors for* *label-free multiplex assays*

*Huan Wang^1, 2^, Lijun Cai^3^, Dagan Zhang^1^, Luoran Shang^4, *^, Yuanjin Zhao^1, 3, 5, *^*

1 Department of Clinical Laboratory, Institute of Translational Medicine, The Affiliated Drum Tower Hospital of Nanjing University Medical School, Nanjing 210002, China

2 The Eighth Affiliated Hospital, Sun Yat-sen University, Shenzhen 518033, China

3 State Key Laboratory of Bioelectronics, School of Biological Science and Medical Engineering, Southeast University, Nanjing 210096, China

4 Shanghai Xuhui Central Hospital, Zhongshan-Xuhui Hospital, and the Shanghai Key Laboratory of Medical Epigenetics, the International Co-laboratory of Medical Epigenetics and Metabolism (Ministry of Science and Technology), Institutes of Biomedical Sciences, Fudan University, Shanghai, 200032, China

5 Chemistry and Biomedicine Innovation Center, Nanjing University, Nanjing 210023, China

Email: [luoranshang@fudan.edu.cn](mailto:luoranshang@fudan.edu.cn); [yjzhao@seu.edu.cn](mailto:yjzhao@seu.edu.cn)

1. **Supplementary Figures and Tables**


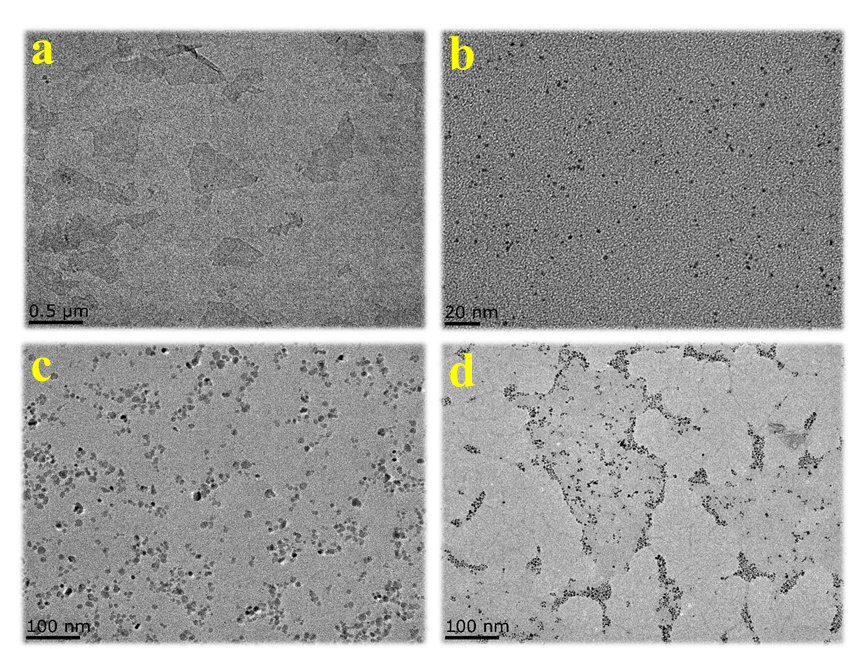


**Fig. S1** TEM characterization. (a) GO sheets. (b) Fe_3_O_4_ NPs. (c) Pt NPs. (d) The NP-loaded GO sheets.


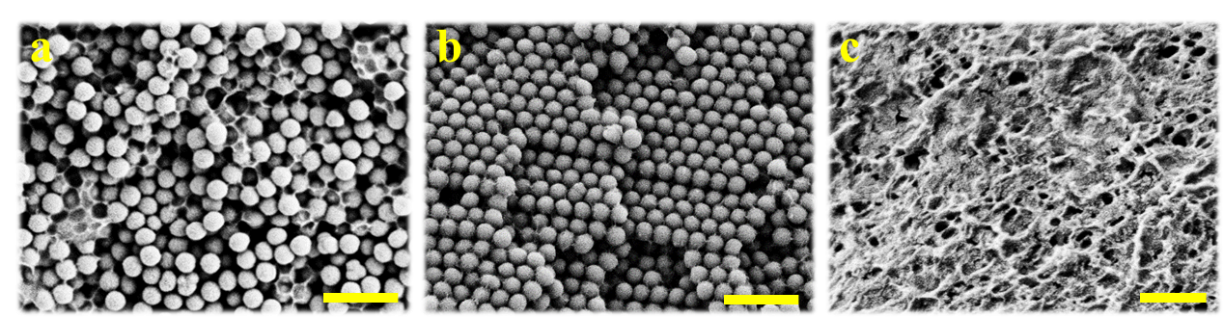


**Fig. S2** SEM characterization of the particles. (a, b) The inner microstructure of the “dark section” (a) and the “photonic section” (b) of the hydrogel composited Janus structural color particle. (c) The collapsed structure of an inverse opal hydrogel particle. Scale bars are 1 μm in a and b, and 500 nm in c.


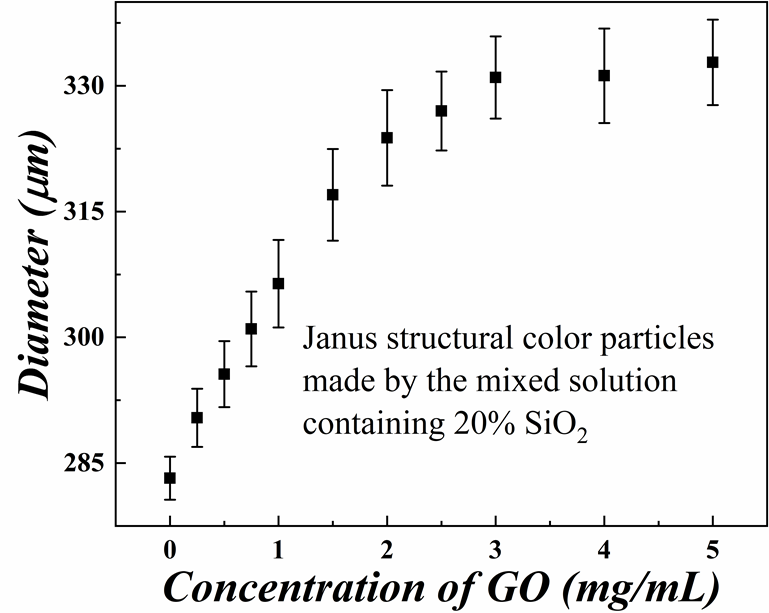


**Fig. S3** Relationships between the diameter of the Janus structural color particles and the concentration of GO of the mixed solution. The concentration of SiO_2_ in the mixed solution was constant (20%). The number of replicates at any concentration was five.


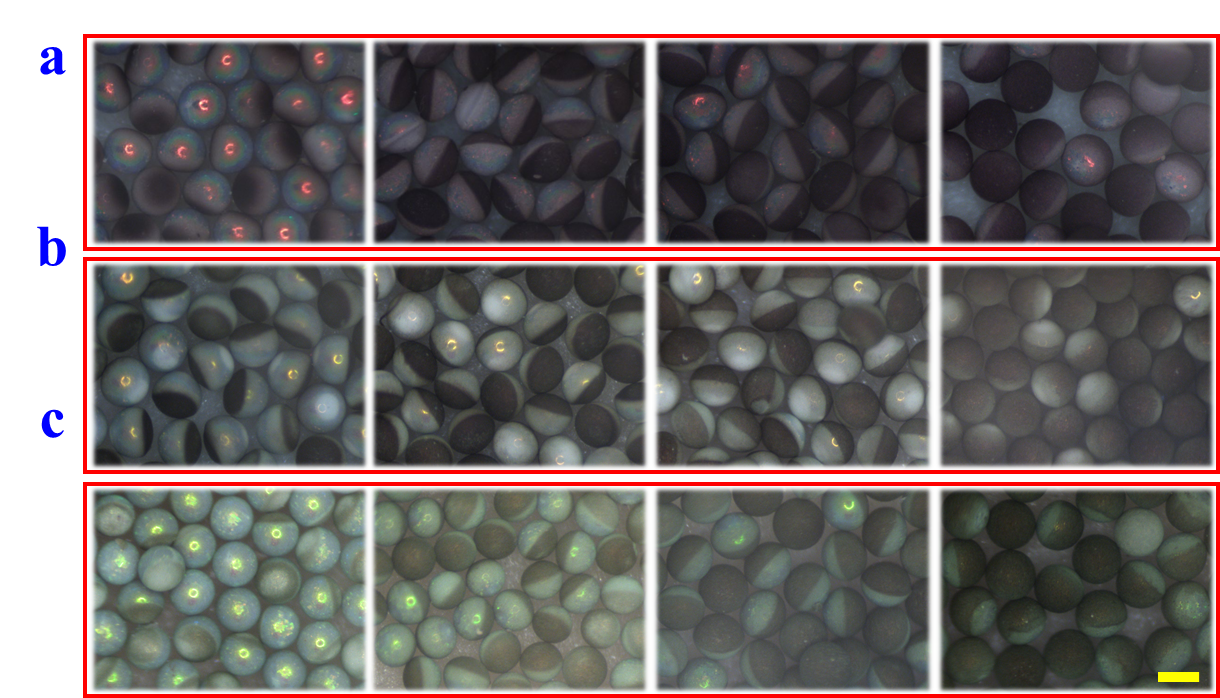


**Fig. S4** Reflection microscope images of Janus structural color particles with different concentration (0.5, 1.5, 2.5, 5 mg/mL) of the GO and colors. The scale bar is 200 μm.


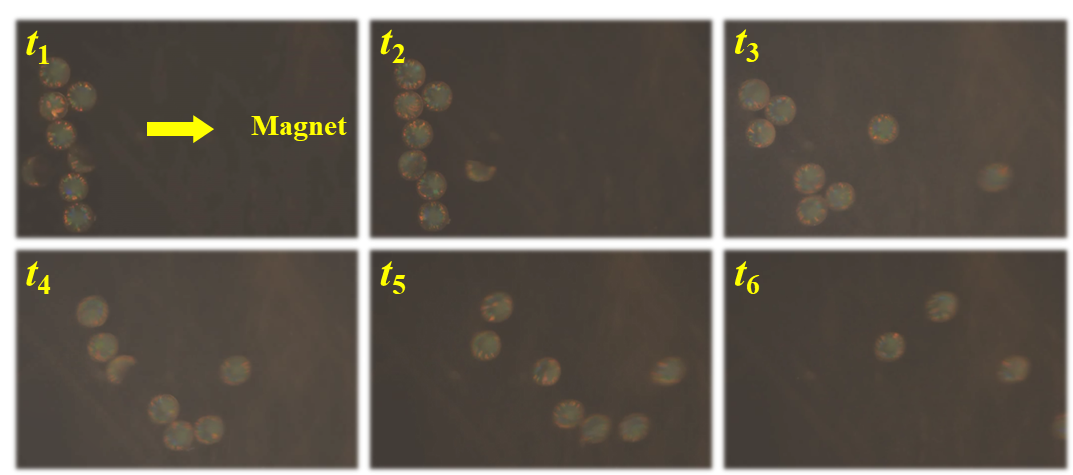


**Fig. S5** Magnetic-induced directional movement of the SCMs under an external magnetic field.


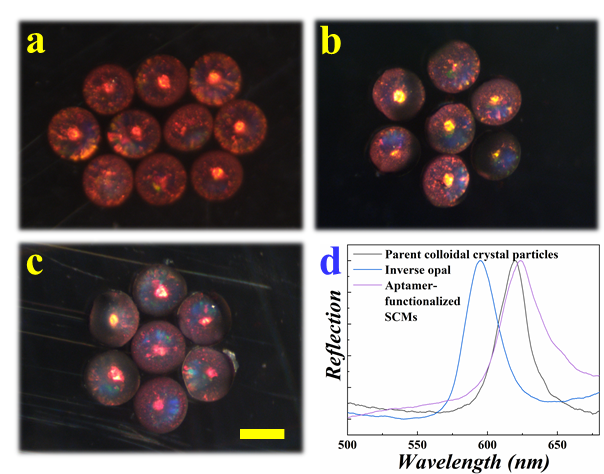


**Fig. S6** (a-c) Reflection image characterization of the parent colloidal crystal particles with orange-red color (a), the corresponding inverse opal particles (b), and the SCMs functionalized with Pb^2+^-responsive aptamers (c). (d) The reflective spectra of these particles.


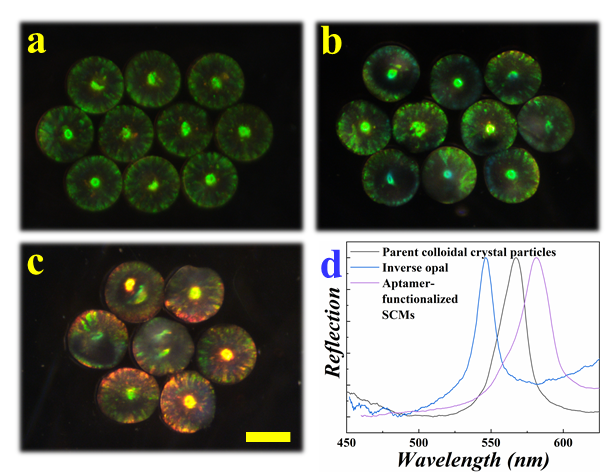


**Fig. S7** (a-c) Reflection image characterization of the parent colloidal crystal particles with green color (a), the corresponding inverse opal particles (b), and the SCMs functionalized with Ag^+^-responsive aptamers (c). (d) The reflective spectra of these particles.


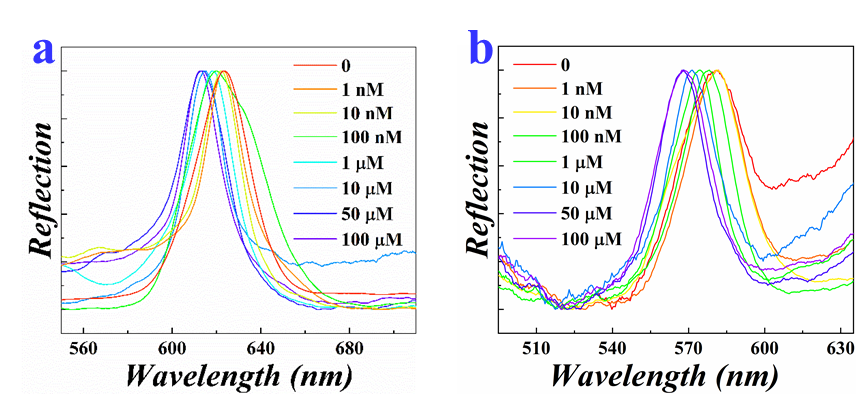


**Fig. S8** (a) Optical response of the SCMs functionalized with Pb^2+^-responsive aptamers incubated in different concentrations of the Pb^2+^ solution. (b) Optical response of the SCMs functionalized with Ag^+^-responsive aptamers incubated in different concentrations of the Ag^+^ solution.


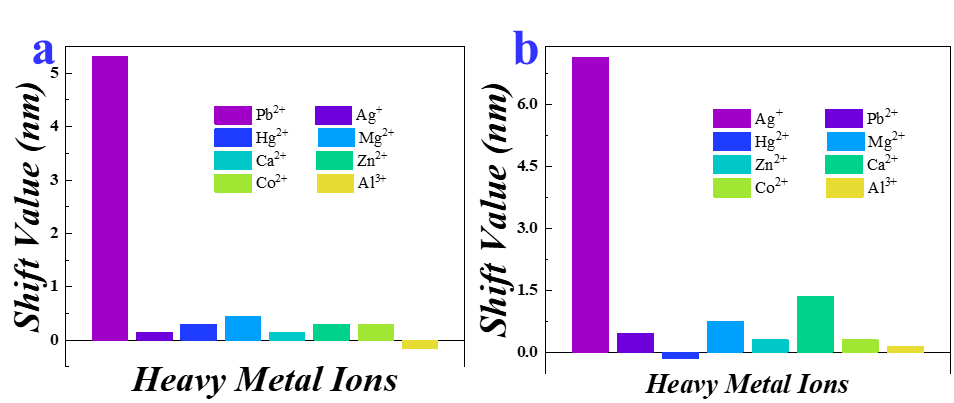


**Fig. S9** The reflection spectra shift of the SCMs functionalized with Pb^2+^-responsive aptamers (a) and Ag^+^-responsive aptamers (c) in various metal ion solutions. (1 μM for target ion and 100 μM for other ions).


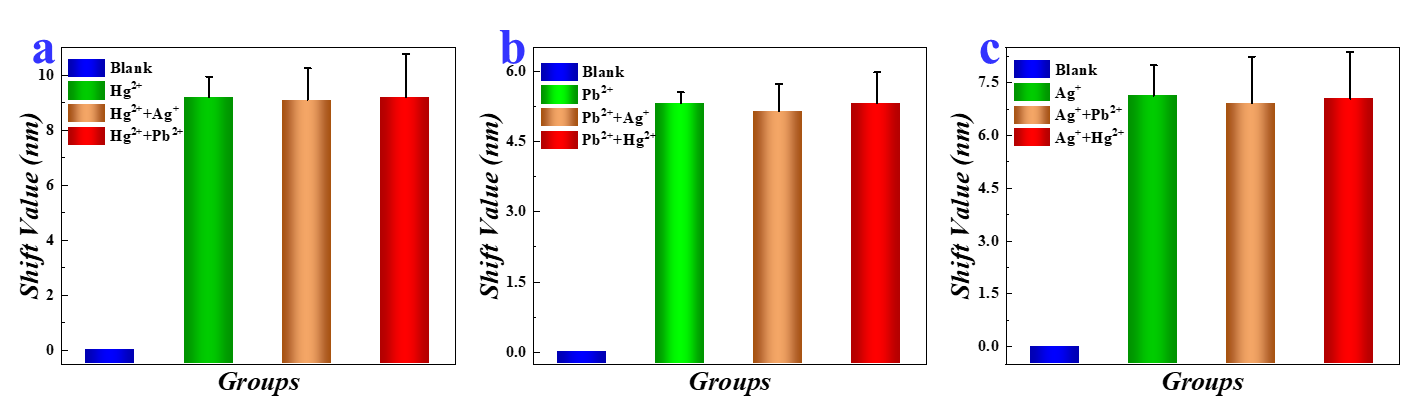


**Fig. S10** The reflection spectra shift of the SCMs modified with Hg^2+^-responsive aptamers (a), Pb^2+^-responsive aptamers (b), and Ag^+^-responsive aptamers (c) in the target low concentration ion solution doped with high concentration other ions. (1 μM for target ion and 100 μM for doped ion). The number of replicates at any concentration was five.

**Table S1** Elemental analysis of the “dark section” of a Janus structural color particle.

| **Element** | **Mass percent (%)** | **Atoms percent (%)** |
| --- | --- | --- |
| C | 24.62 | 37.16 |
| O | 43.07 | 48.80 |
| Si | 19.76 | 12.75 |
| Fe | 0.53 | 0.17 |
| Pt | 12.02 | 1.12 |
| Total | 100 | 100 |

**Table S2** Sequences for the DNA aptamers.

| **Aptamer** | **Sequences** |
| --- | --- |
| Aptamer 1 | 5’-NH_2_-(CH_2_)_6_-TTCTTTCTTCCCCTTGTTTGTT-(CH_2_)_6_-NH_2_-3’ |
| Aptamer 2 | 5’-NH_2_-(CH_2_)_6_-GGTTGGTGTGGTTGG-(CH_2_)_6_-NH_2_-3’ |
| Aptamer 3 | 5’-NH_2_-(CH_2_)_6_-CTCTCTTCTCAAAAAACACAACACAC-(CH_2_)_6_-NH_2_-3’ |

1. **Description of the Supplementary Movies**

**Supplementary Movie. S1.** SCM in H_2_O_2_ environment.

**Supplementary Movie. S2.** SCM movement under magnet guidance.

**Supplementary Movie. S3.** SCM in H_2_O_2_ environment with magnet guidance.

**Supplementary Movie. S4.** SCMs in H_2_O_2_ environment for multiple detection.
